# Radiation-induced VEGF-C expression and endothelial cell proliferation in lung cancer

**DOI:** 10.1007/s00066-014-0708-z

**Published:** 2014-07-03

**Authors:** Yu-Hsuan Chen, Shiow-Lin Pan, Jing-Chi Wang, Sung-Hsin Kuo, Jason Chia-Hsien Cheng, Che-Ming Teng

**Affiliations:** 1 Department of Oncology, National Taiwan University Hospital, Taipei, Taiwan; 2 Pharmacological Institute, College of Medicine, National Taiwan University, No. 7 Chung-Shan South Road, 100 Taipei, Taiwan; 3 Graduate Institute of Clinical Medicine, National Taiwan University College of Medicine, Taipei, Taiwan; 4 Department of Internal Medicine, National Taiwan University College of Medicine, Taipei, Taiwan

**Keywords:** Radiation, Vascular endothelial growth factor C, Lung cancer cells, Endothelial cells, PI3K/Akt/mTOR signaling pathway, Strahlung, Vaskulärer endothelialer Wachstumsfaktor C, Lungenkrebszellen, Endothelzellen, PI3K/Akt/mTOR-Signalweg

## Abstract

**Background:**

The present study was undertaken to investigate whether radiation induces the expression of vascular endothelial growth factor C (VEGF-C) through activation of the PI3K/Akt/mTOR pathway,subsequently affecting endothelial cells.

**Materials and methods:**

Radiotherapy-induced tumor micro-lymphatic vessel density (MLVD) was determined in a lung cancer xenograft model established in SCID mice. The protein expression and phosphorylation of members of the PI3K/Akt/mTOR pathway and VEGF-C secretion and mRNA expression in irradiated lung cancer cells were assessed by Western blot analysis, enzyme-linked immunosorbent assays (ELISAs), and reverse transcriptase–polymerase chain reaction (RT-PCR). Moreover, specific chemical inhibitors were used to evaluate the role of the PI3K/Akt/mTOR signaling pathway. Conditioned medium (CM) from irradiated control-siRNA or VEGF-C-siRNA-expressing A549 cells was used to evaluate the proliferation of endothelial cells by the MTT assay.

**Results:**

Radiation increased VEGF-C expression in a dose-dependent manner over time at the protein but not at the mRNA level. Radiation also up-regulated the phosphorylation of Akt, mTOR, 4EBP, and eIF4E, but not of p70S6K. Radiation-induced VEGF-C expression was down-regulated by LY294002 and rapamycin (both *p* < 0.05). Furthermore, CM from irradiated A549 cells enhanced human umbilical vein endothelial cell (HUVEC) and lymphatic endothelial cell (LEC) proliferation, which was not observed with CM from irradiated VEGF-C-siRNA-expressing A549 cells.

**Conclusions:**

Radiation-induced activation of the PI3K/Akt/mTOR signaling pathway increases VEGF-C expression in lung cancer cells, thereby promoting endothelial cell proliferation.

## Abstract (de)

**Hintergrund und Ziel:**

Die vorliegende Studie untersucht, ob die Strahlung die Expression von VEGF-C (vascular endothelial growth factor C) mittels Aktivierung des PI3K/Akt/mTOR-Signalwegs induziert und anschließend die endothelialen Zellen beeinflusst.

**Methoden und Material:**

Die durch Strahlentherapie induzierte Mikrolymphgefäßdichte (MLVD) im Tumor wurde in einem Lungenkrebs-Xenograftmodell mit SCID-Mäusen ermittelt. Die Proteinexpression, die Phosphorylierung der Mitglieder des PI3K/Akt/mTOR-Signalwegs und die Expressionder VEGF-C-Sekretion und mRNA-Expression in bestrahlten Lungenkrebszellen wurden jeweils mittels Westernblot-Analyse, ELISA (enzyme-linked immunosorbent assays) und RT-PCR (Reverse-Transkriptase-Polymerasekettenreaktion)bewertet. Außerdem wurden spezifische chemische Inhibitoren verwendet, um die Rolle des PI3K/Akt/mTOR-Signalwegs auszuwerten. Konditioniertes Medium (CM) aus bestrahlten Kontroll-siRNA-exprimierenden und ausunbestrahlten VEGF-C-siRNA-exprimierenden A549-Zellen wurde benutzt, um die Proliferation von Endothelzellen mittels MTT-Assay zu bewerten.

**Ergebnisse:**

Strahlung erhöht die dosisabhängige und zeitabhängige VEGF-C-Expression auf Protein- anstatt auf mRNA-Ebene. Durch die Bestrahlung wurde die Phosphorylierung von Akt, mTOR, 4EBP und eIF4E hochreguliert, nicht jedoch von p70S6K. Die trahleninduzierte VEGF-C-Expression wurde durch LY294002 und Rapamycin herunterreguliert (beide *p* < 0,05). Des Weiteren fördert CM aus den unbestrahlten A549-Zellen die Proliferation der menschlichen Nabelvenenendothelzellen (HUVECs) und der lymphatischen Endothelzellen (LECs). CM aus den unbestrahlten und VEGF-C-iRNA-exprimierenden A549-Zellen hat jedoch keine proliferationsfördernde Wirkung.

**Schlussfolgerungen:**

Die strahleninduzierte Aktivierung des PI3K/Akt/mTOR-Signalwegs kann die Expression des VEGF-C in Lungenkrebszellen erhöhen und die Proliferation der Endothelzellen fördern.

## Introduction

About 1.2 million new cases of lung cancer occur annually worldwide. Approximately 35 % of patients with non-small cell lung cancer (NSCLC) are diagnosed with stage III disease [1]. Of the stage III subgroup, only about 20 % of patients can be treated with surgery; the majority of patients may choose radiotherapy (RT) for local treatment, and meta-analysis indicates that combined chemotherapy and radiotherapy (CCRT) has become the standard of care for this group of patients, with good outcomes [2]. Specifically, among patients who underwent CCRT with a standard dose of radiation (> 60 Gy), the complete remission rate ranged from 10 to 60 %, with a 5-year survival rate of 5–23 % [3]. Patients surviving for 6–12 months had a statistically significantly increased survival rate when the intrathoracic tumor was controlled. Patients treated with 50–60 Gy showing tumor control had a 3-year survival rate of 22 %, compared with 10 % for patients with intrathoracic failure [4], suggesting that local intrathoracic control may be predictive of patient survival. Therefore, it is important to enhance the radiosensitivity of lung cancer cells, which may affect local control and subsequent survival. In this study, we attempted to analyze the environmental changes after RT that increase the likelihood of the resistance of cancer cells to RT.

Blood and lymphatic vascular system development and maintenance are coupled through multiple growth factors and receptors expressed on blood and lymphatic endothelial cells (BECs and LECs, respectively). Vascular endothelial growth factors A and C (VEGF-A and VEGF-C, respectively) are two major growth factors that promote endothelial cell proliferation, migration, and induction of permeability. VEGF-C binding to VEGF receptor 3 (VEGFR-3) induces LEC proliferation and lymphatic vascular hyperplasia [5, 6]. VEGF-C can also bind to VEGFR-2, eliciting a response that is similar to that induced by VEGF-A but less potent [7].

The expression of VEGF-C and VEGFR-3 in lung cancer tumor cells was significantly associated with more advanced regional lymph node metastasis [8–10]. Previous studies also demonstrated that radiation not only induced angiogenesis but also caused lymphangiogenesis [11]. For example, Nathanson et al. reported that subtherapeutic hyperthermia or radiation increased lymphatic flow from tumors, which was associated with an increase in metastasis of melanoma [12]. Talmadge et al. also showed that sublethal dose irradiation activated genes or signal pathways that induced functional changes that increased the survival or metastasis of cancer cells [13]. Moreover, radiation of tumor cells induced vascular or microenvironment changes that enhanced capillary invasion capacity [14, 15]. In the present study, we investigated the mechanism and consequences of irradiation-induced VEGF-C expression in lung cancer cells.

## Materials and methods

### Lung cancer cells

The human lung cell lines A549 and H1299 were obtained from the American Type Culture Collection (Manassas, Va.) and were maintained in Dulbecco’s modified Eagle medium (DMEM) or RPMI supplemented with nonessential amino acids, l-glutamine, a twofold vitamin solution, sodium pyruvate, 10 % fetal bovine serum, penicillin, and streptomycin. Cells were cultured at 37 °C in a humidified atmosphere of 5 % CO_2_ and 95 % air.

### Establishment of lung cancer xenografts and irradiation

Male SCID mice (5–6 weeks old) were obtained from the National Taiwan University Animal Center, Taipei, Taiwan. To establish the xenografts, 1 × 10^6^ A549 cells were injected subcutaneously into the right hind limb. At 10 days postimplantation, mice were immobilized in a customized harness that exposed the right hind leg. The remainder of the body was shielded by five times the half-value thickness of lead. A cobalt-60 unit was used to irradiate the primary tumor with 25 or 50 Gy (5-Gy or 10-Gy daily fractions, at a dose rate of 1 Gy/min).

On postimplantation day 9, tumor volumes were calculated every 3 days using a standard formula: width^2^ × length/2. Tumors were harvested at the time of sacrifice 14 days after completion of the radiotherapy and fixed in 10 % neutral buffered formalin and processed for immunohistochemical evaluations. All mice were group-housed under a fixed light–dark cycle (12 h of light and 12 h of darkness) with ad libitum access to sterilized food and water. All experimental procedures were performed in accordance with protocols approved by the Committee of Experimental Animal Management at the College of Medicine, National Taiwan University.

### Immunohistochemistry analysis

All tissues were fixed in 10 % neutral buffered formalin and embedded in paraffin using standard protocols. Immunohistochemistry (IHC) analysis was performed on a single representative block from each case. Tissue sections (5 μm) were dewaxed, and antigen retrieval was performed in a citrate buffer (pH 6) using an electric pressure cooker set at 120 °C for 5 min as previously described (Choi et al. 2005). Sections were incubated for 5 min in 3 % hydrogen peroxide to quench endogenous tissue peroxidase. The sections were incubated in antibodies specific for the lymphatic endothelial marker, D2-40 antibodies (1:200 dilution; Biocompare, San Francisco, Calif.), for 60 min at room temperature. After washing unbound primary antibody, sections were treated with biotinylated secondary antibodies followed by avidin coupled to biotinylated HRP at room temperature according to the manufacturer’s instructions (DAKO, Carpinteria, Calif.). Immunohistochemical reactions were developed with diaminobenzidine as the chromogenic peroxidase substrate. Sections were counterstained with hematoxylin. Specificity was verified by negative control reactions that did not contain primary antibodies as well as by appropriate cytoplasmic reactions for each antigen in positive control tissues.

### Microlymphatic vessel density assessment

Immunohistochemical reactions for D2-40 were analyzed at low magnification (× 40), and lymphatic vessels were counted in five representative high-magnification (× 400; 0.152 mm^2^; 0.44-mm diameter) fields. Single immunoreactive lymphatic endothelial cells, or lymphatic endothelial cell clusters separate from other microlymphatic vessels, were counted as individual microlymphatic vessels. The mean visual microlymphatic vessel density (MLVD) for D2-40 was calculated.

### Culture of HUVECs and LECs

Human umbilical vein endothelial cells (HUVECs) were purchased from the Food Industry Research and Development Institute and cultured in M199 medium (Life Technologies, Carlsbad, Calif.) supplemented with 20 % fetal bovine serum, endothelial cell growth supplement (Millipore, Billerica, Mass.), heparin, l-glutamine, penicillin, and streptomycin. Human lymphatic endothelial cell (LECs) were isolated from human lymph nodes (ScienCell, Carlsbad, Calif.) and cultured in endothelial cell medium (ScienCell). The cells were cultured at 37 °C in a humidified atmosphere of 5 % CO_2_ and 95 % air.

### Irradiation protocol

Cell culture plates were irradiated with different doses of radiation (0–10 Gy) using a cobalt-60 unit. The source–skin distance technique was set at 80 cm at the bottom of the flask. The dose rate was 1 Gy/min. Dosimetry was performed with an ionization chamber.

### Western blot analysis

After cells were treated with various radiation doses and drugs, total protein was collected at different times. Protein was extracted using Mammalian Protein Extraction Reagent (M-PER; Pierce, Rockford, Ill.). An equal amount of protein from each cell line was loaded per lane and separated on a 10 % sodium dodecyl sulfate (SDS)–Tris glycine polyacrylamide gel electrophoresis (PAGE) gel. Gels were transferred onto nitrocellulose membranes (Novex, San Diego, Calif.) and blocked overnight by incubating with 1 × Tris-buffered saline containing 0.1 % Tween and 5 % nonfat dry milk. Membranes were probed with antibodies against phospho-Akt (p-Akt; Cell Signaling, Beverly, Mass.), phospho-mTOR (p-mTOR; Novus International, St. Louis, Mo.), phospho-eIF4E (p-4EBP), phospho–p70 ribosomal protein S6 kinases (p-p70S6K; Cell Signaling), phospho–eukaryotic translation initiation factor 4E (p-eIF4E; Cell Signaling), VEGF-C (Zymed Laboratories, South San Francisco, Calif.), Akt, mTOR, 4EBP, eIF4E, and actin (all from Chemicon International, Temecula, Calif.). Bound antibodies were then detected using the appropriate peroxidase-coupled secondary antibodies, followed by incubation with an enhanced chemiluminescence detection system (ECL; Boehringer Mannheim, Mannheim, Germany).

### Enzyme-linked immunosorbent assay to detect secreted VEGF-C

Cell culture supernatant was collected after culturing A549 cells in completed culture medium for 24 h after irradiation. VEGF-C secretion was measured in the cell culture supernatant using a commercial enzyme-linked immunosorbent assay (ELISA) kit (R&D Systems, Minneapolis, Minn.) according to the manufacturer’s protocol. Color intensity was measured by a plate reader at 450 nm. Data represent the average of three different assays.

### Reverse transcription–polymerase chain reaction

Reverse transcription (RT) of RNA was performed in a final reaction volume of 20 µl containing 5 µg of total RNA in Moloney murine leukemia virus (MMLV) reverse transcriptase buffer that contained 10 mM dithiothreitol, dNTPs (2.5 mM each), 1 mM (dT) primer, and 200 U of MMLV reverse transcriptase (Promega, Madison, Wis.). The reaction mixture was incubated at 37 °C for 2 h, and was terminated by heating at 70 °C for 10 min. A portion of the reaction mixture was then amplified by PCR with the following primer pairs: VEGF-C, sense 5′-CAGTTACGGTCTGTGTCCAGTGTAG-3′ and antisense 5′-GGACACACATGGAGGTTTAA-3′; β-actin, sense 5′- ATCCGCAAAGACCTGTACGC-3′ and antisense 5′-TGTGTGGACTTGGGAGAGGA-3′. A total of 30 cycles were performed. The products were separated on 2 % agarose gels, stained with 1 mg/ml ethidium bromide, and visualized using a UVP GDS-7900 digital imaging system. The results were confirmed by conducting at least three independent experiments.

### Transfection of small interfering RNA

Small interfering RNA (siRNA) duplexes specific for VEGF-C or Akt were purchased from Santa Cruz Biotechnology (Santa Cruz, Calif.); control siRNAs with sequences that do not target any gene product were obtained from Invitrogen (Carlsbad, Calif.). Cells were transfected with 25 nM VEGF-C siRNA, Akt siRNA, or control siRNA in serum-free Opti-MEM using the oligofectamine method (Invitrogen) for 1 h at 37 °C. After changing the culture medium, cells were cultured for 24 h at 37 °C prior to further experiments.

### Conditioned media collection

A549 (1 × 10^5^ cells) cells expressing control or VEGF-C siRNA were cultured in a 6-cm dish overnight, washed two times with PBS, and then incubated in medium before exposure to 5-Gy irradiation. Conditioned medium (CM) was harvested at 24 h after irradiation. The origin of CM was designated as the following: (1) non-irradiated A549 CM, (2) irradiated A549 CM, (3) irradiated VEGF-C-siRNA A549 CM, and (4) irradiated control-siRNA A549 CM.

### Cell proliferation assay

Cells were cultured in 96-well plates at a density of 5,000 cells/well. The quantity of viable cells was estimated by a colorimetric assay using 3-(4,5-dimethylthiazol-2-yl)-2,5-diphenyltetrazolium bromide (MTT). MTT (10 µl of 5 mg/ml solution, Sigma, Germany) was added to each well and incubated for 4 h at 37 °C. The cells were then treated with 40 µl/well dimethyl sulfoxide (DMSO) and incubated for 60 min at 37 °C. The absorbance of each well was determined in an ELISA plate reader using an activation wavelength of 570 nm and a reference wavelength of 630 nm. The percentage of viable cells was determined by comparison with untreated control cells.

### Statistical analysis

All data presented are the mean ± standard deviation of experiments repeated three or more times. A paired t-test was used to evaluate statistically significant differences between indicated groups in specified tests; *p* values < 0.05 were considered statistically significant.

## Results

### Sublethal RT dose significantly increased tumor MLVD in SCID mice bearing ectopic lung cancer xenografts

**Fig. 1:**
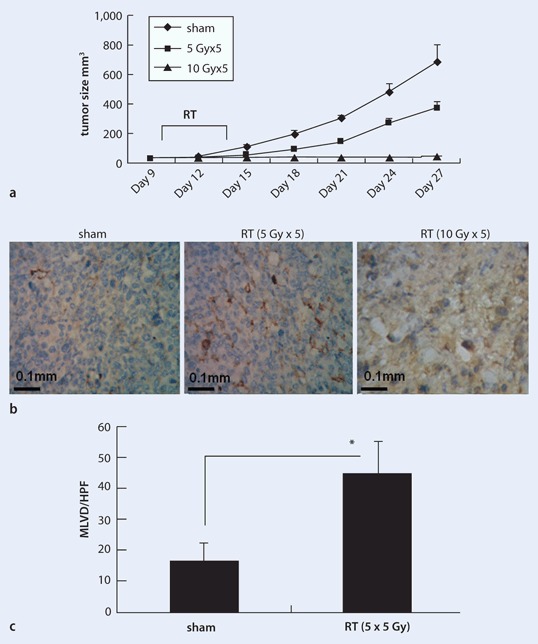
**a–c** Effects of radiotherapy (*RT*) on tumor lymphatic vessel density. A549 cells were seeded in the right legs of SCID mice. **a** Tumor sizes were measured every 3 days. On day 10, tumors were exposed to sham or to irradiation with 5 Gy × 5 or 10 Gy × 5. Data represent the tumor growth curve. **b** The tumors were removed 14 days after RT, and tumor sections from all the groups were stained with an antibody specific for D2-40. The images are representative of three independent experiments. **c** The mean microlymphatic vessels density (*MLVD*) was counted in ten representative high-power (× 200) fields in each group. (*p < 0.05)

As shown in Fig. 1a, 5 fractions of 10 Gy significantly suppressed the tumor growth, and 5 fractions of 5 Gy had a mild therapeutic effect. Analysis of tumor MLVD by examining the expression of the lymphatic endothelium vessel-specific marker D2-40 revealed stronger expression in the tumors irradiated with 5 fractions of 5 Gy (Fig. 1b). However, in tumors irradiated with 5 fractions of 10 Gy, severe tissue necrosis was observed (Fig. 1b). As shown in Fig. 1c, the MLVD was significantly higher in tumors irradiated with 5 fractions of 5 Gy. However, no MLVD was found in severe tissue necrosis of tumors irradiated with 5 fractions of 10 Gy.

### Radiation induced a dose-dependent increase in VEGF-C expression by lung cancer cells

**Fig. 2:**
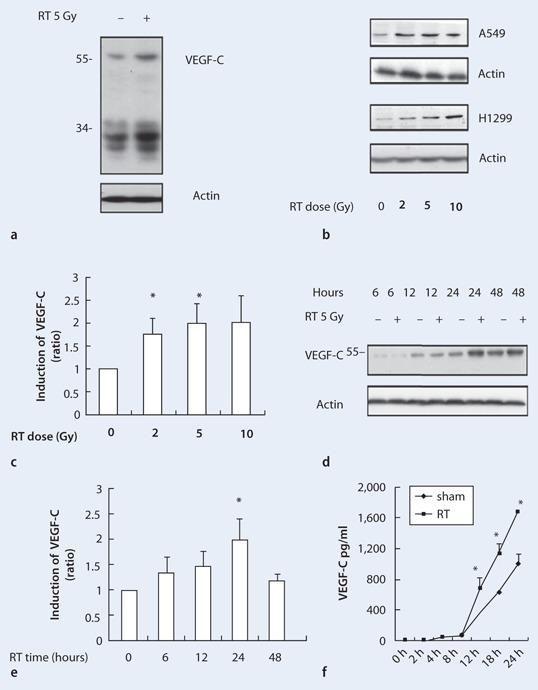
**a–f** Radiation increases VEGF-C expression in lung cancer cells. **a** VEGF-C expression, including a 58-kDa VEGF-C propeptide, as well as the 31- and 29-kDa secreted peptides, increased after 5 Gy of irradiation as compared with the non-irradiated group. **b** The effect of radiation-induced VEGF-C expression was dose-dependent in A549 and H1299 cell lines. **c** Quantification of the immunoblot data from A549 cells. **d** The expression of VEGF-C increased over time in A549 cells. **e** The immunoblot data in **d** were quantified. **f** VEGF-C secretion into the cell culture supernatant was determined by ELISA. Data are presented as mean ± SE, *n* = 3. (*p < 0.05 compared with non-irradiated cells)

Due to the proteolytic processing of VEGF-C in cancer cells, a 58-kDa VEGF-C propeptide as well as 31- and 29-kDa secreted VEGF-C peptide levels were evaluated by Western blot analysis [16]. As compared with the non-irradiated cells, those treated with 5-Gy irradiation for 24 h had increased VEGF-C expression in A549 cells (Fig. 2a). In A549 and H1299 lung cancer cells, radiation increased VEGF-C expression in a dose-dependent manner (Fig. 2b). At 24 h after exposure to radiation, VEGF-C expression increased 1.75-, 1.98-, and 2.01-fold with 2.5, 5, and 10 Gy, respectively, relative to the non-irradiated group in A549 cells (*p* < 0.05 for cells treated with 2 and 5 Gy; Fig. 2c). In addition, the effects of 5-Gy irradiation on VEGF-C expression increased over time relative to the non-irradiated control cells (Fig. 2d and e). Moreover, assessment of the soluble VEGF-C peptides by ELISA and Western blot analysis revealed their up-regulation after radiation treatment over time (Fig. 2f).

### Radiation activates the Akt/mTOR/4EBP/eIF4E signaling pathway in the A549 lung cancer cell line

**Fig. 3:**
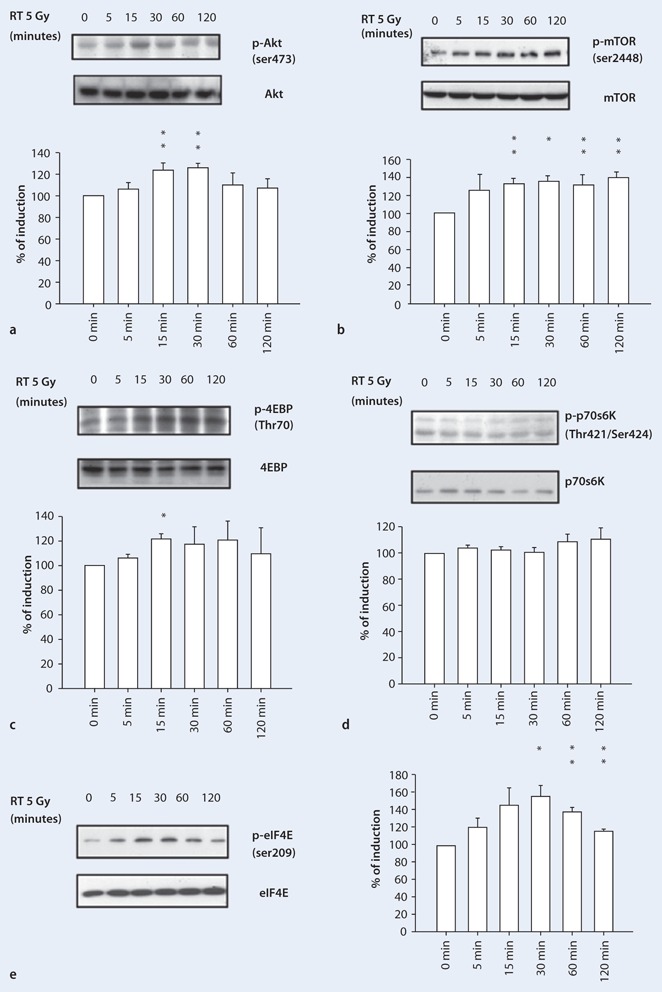
**a–e** Radiation up-regulated phosphorylation of the Akt/mTOR/4EBP/eIF4E pathway. **a** Radiation induced phosphorylation of Akt over time, peaking at 15 min postirradiation. **b** mTOR, **c** 4EBP, and **e** eIF4E phosphorylation levels increased after irradiation. **d** p-p70S6K was not induced by radiation. Data are presented as mean ± SE, *n* = 3. (*p < 0.05; **p < 0.01)

The effects of radiation on the PI3K/Akt/mTOR signaling pathway were determined by Western blot analysis. After 5-Gy radiation, protein samples were collected at 5, 15, 30, 60, and 120 min. Radiation-induced Akt phosphorylation was significantly increased by 1.2-fold from the control at 15 min (*p* < 0.01; Fig. 3a). p-mTOR levels were up-regulated 1.2- to 1.4-fold after 5 and 15 min of irradiation, respectively, and this effect persisted for 120 min (Fig. 3b). p-4EBP, a downstream signaling target of mTOR, was also significantly increased at 15 min (Fig. 3c); however, p-p70S6K was not induced by radiation (Fig. 3d). Additionally, p-eIF4E gradually increased from 5 min after irradiation, peaking at 1.5-fold at 30 min (Fig. 3e). Taken together, these results demonstrated that radiation up-regulated the phosphorylation of Akt, mTOR, 4EBP, and eIF4E, but not of p70S6K.

### Irradiation-induced VEGF-C expression was mediated through PI3K/Akt/mTOR signaling in the A549 lung cancer cell line

**Fig. 4:**
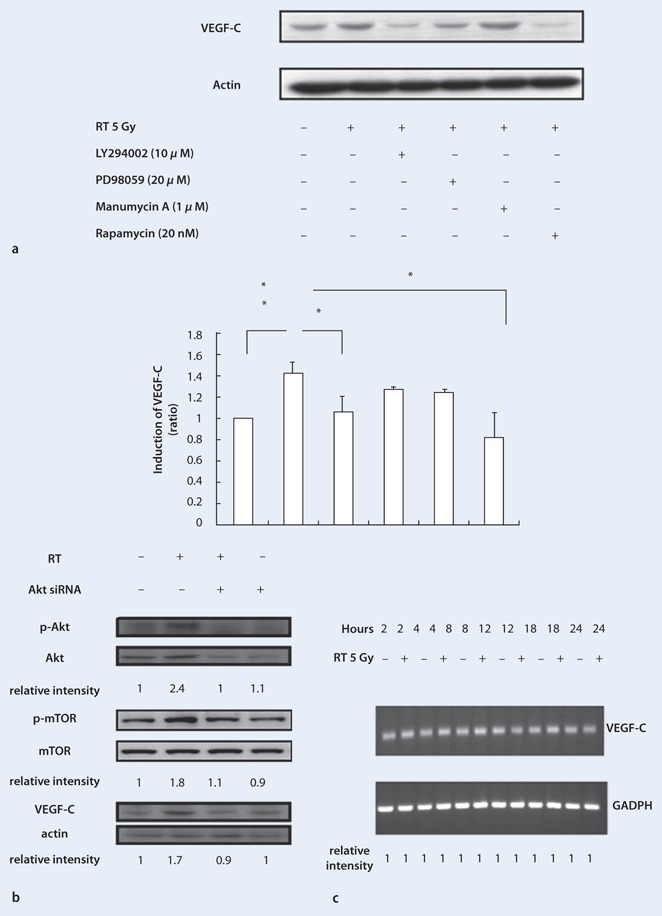
**a–c** Radiation-induced VEGF-C expression is mediated by PI3K/Akt/mTOR signal transduction. **a** Radiation-induced VEGF-C expression was significantly reduced by LY294002 and rapamycin as determined by quantification of the immunoblot data. **b** Radiation-induced Akt and mTOR phosphorylation and VEGF-C expression was inhibited by Akt siRNA. **c** Radiation-induced VEGF-C expression was not at the transcriptional level as determined by RT-PCR analysis of VEGF-C mRNA. Data are presented as mean ± SE, *n* = 3. (*p < 0.05; **p < 0.01 compared with untreated cells)

To investigate the relationship between radiation, the PI3K/Akt/mTOR signaling pathway, and VEGF-C expression, we used 10 µM LY294002 as a PI3K/Akt inhibitor, 20 μM PD98059 as an extracellular signal-regulated kinase (ERK) inhibitor, 1 μM manumycin A as a P38 inhibitor, and 20 nM rapamycin as an mTOR inhibitor. A549 cells were pretreated with these inhibitors 1 h before 5-Gy irradiation. As shown in Fig. 4a, radiation-induced VEGF-C expression was significantly reduced by LY294002 and rapamycin (both *p* < 0.05), suggesting that PI3K/Akt and mTOR signaling were required for irradiation-induced VEGF-C expression.

To confirm the relationship between PI3K/Akt and mTOR as well as the role of Akt in irradiation-induced VEGF-C expression, we manipulated Akt expression using Akt siRNA. As shown in Fig. 4b, Akt phosphorylation was augmented by irradiation and blocked by Akt siRNA. Furthermore, irradiation-induced mTOR phosphorylation and VEGF up-regulation in A549 cells were inhibited by Akt siRNA (Fig. 4b), indicating that PI3K/Akt/mTOR signaling mediates irradiation-induced VEGF-C expression.

Analysis of VEGF-C mRNA expression by RT-PCR revealed no obvious changes in VEGF-C mRNA expression between the sham and irradiated groups (Fig. 4c), indicating that the effects of irradiation on VEGF-C expression were at the translational level, but not the transcriptional level.

### Irradiation-induced VEGF-C expression promotes the proliferation of HUVECs and LECs

**Fig. 5:**
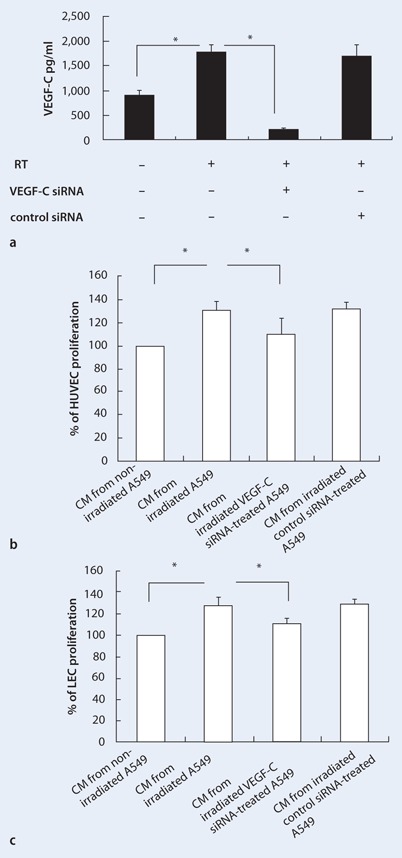
**a–c** Radiation-induced VEGF-C promotes HUVEC and LEC proliferation. **a** VEGF-C in the cell culture supernatant as determined by ELISA. **b, c** The conditioned media (*CM*) from irradiated A549 cells also promoted HUVEC and LEC proliferation, which was significantly suppressed when they were cultured in CM from irradiated A549 cells expressing VEGF-C-siRNA. Data are presented as mean ± SE, *n* = 3. (*p < 0.05)

The effect of irradiation-induced VEGF-C expression on HUVEC and LEC proliferation was assessed using siRNA specific for VEGF-C. The inhibition of VEGF-C siRNA on the VEGF-C levels in the cell culture supernatant was first confirmed by ELISA. As shown in Fig. 5a, VEGF-C, but not control siRNA, significantly reduced the irradiation-induced VEGF-C expression in A549 cells.

The effect of CM on HUVEC and LEC proliferation was next determined using the MTT assay. As shown in Fig. 5b, HUVEC proliferation was significantly increased by 1.30-fold after culturing HUVECs in CM from irradiated A549 cells as compared with that from non-irradiated A549 cells (*p* < 0.01). This effect was significantly inhibited when HUVECs were cultured in CM from VEGF-C siRNA-expressing A549 cells (*p* = 0.05; Fig. 5b). The CM from irradiated A549 cells also increased LEC proliferation by 1.28-fold, which was significantly suppressed when LECs were cultured in CM derived from irradiated VEGF-C-siRNA-expressing A549 cells (*p* = 0.03; Fig. 5c).

## Discussion

Tumor cells can produce multiple growth factors to create a microenvironment conducive for BEC and LEC growth. Tumor cell progression and metastasis may benefit from cross-talk between tumor cells, VECs, and LECs [17, 18]. Radiation has antiangiogenic as well as antilymphogenic effects by damaging cell membranes, DNA, and endothelial cells, resulting in apoptosis. However, radiation also has proangiogenic and prolymphogenic effects either through direct mechanisms or through signals that are released by irradiated cancer cells and the tumor microenvironment. Recent studies also demonstrated a close association between radiosensitivity, proangiogenic growth factors, and endothelial cell survival; VEGFR inhibitors enhance the therapeutic efficacy of irradiation in NSCLC by hindering the repair of sublethal radiation damage [19].

In the current study, radiation increased VEGF-C expression in a dose-dependent manner over time. Furthermore, CM from irradiated A549 cells enhanced proliferation of HUVECs and LECs, which was ameliorated in irradiated A549 cells expressing VEGF-C siRNA, suggesting that VEGF-C may play an important role in the angiogenic or lymphogenic effect of radiation in lung cancer. These results were consistent with those of other studies, which reported that radiation can both stimulate and inhibit angiogenesis through regulation of the proangiogenic and antiangiogenic balance [20, 21]. For example, Sonveaux et al. [20] found that irradiation stimulated migration and tubulogenesis of HUVECs accompanied by up-regulation of endothelial nitric oxide synthase (eNOS). In human tumor samples, the clinical relevance of irradiation-induced angiogenic growth factor expression has been observed [21]. For example, in a study of rectal cancer patients receiving neoadjuvant RT followed by surgery, a significant increase in VEGF expression was observed in postradiation specimens compared with primary tumor samples [21]. Therefore, radiation might induce a defense mechanism against radiation damage through the activation of a potential proangiogenic reaction. Most studies demonstrated radiation-induced VEGF-A expression but the novel finding in our study is VEGF-C over-expression after irradiation in lung cancer cells.

Several studies have demonstrated that radiation can activate various signaling pathways, including the PI3K, MAPK, JNK, and NF-κB signaling pathways [22, 23]. The PI3K/Akt/mTOR signaling pathway is involved in tumorigenesis, translation of proteins for cell cycle progression, and lung cancer cell proliferation [24, 25]. In addition to killing tumor cells, radiation could induce radioprotective signaling. Several studies analyzing the mechanism of radioresistance have demonstrated that it may be associated with activation of the PI3K/Akt/mTOR pathway [26, 27]. Sunavala-Dossabhoy et al. [26] observed that radiation increased the expression of a radioprotective protein, Tousled-like kinase 1B (TLK1B), which was preceded by an increase in p-Akt and p-4EBP. Irradiation also induced phosphorylation of Akt and mTOR in a breast cancer cell line, which was attenuated by mTOR and PI3K inhibitors. These findings suggest that the mTOR inhibitor attenuates radiation-induced Akt/mTOR prosurvival signaling and enhances the cytotoxic effects of radiation in breast cancer cells [27]. Moreover, Dionysopoulos et al. [28] demonstrated that clinicopathological parameters as well as high mTOR and CCND1 mRNA expression were associated with poor patient outcome in localized laryngeal cancer. The present study further supported that radiation can induce phosphorylation of Akt, mTOR, 4EBP, and eIF4E, indicating that the PI3K/Akt/mTOR/eIF4E signaling pathway can be activated by irradiation.

mTOR, p38, and JNK play important roles in the up-regulation of VEGF-C expression, and inhibition of mTOR by rapamycin has an anti-lymphangiogenic effect, which inhibited lymphatic metastasis [29]. Also, activation of the PI3k/Akt signaling pathway up-regulated VEGF-C expression [30]. These observations were supported by the present study, which found down-regulation of radiation-induced VEGF-C expression after inhibition of PI3K/AKT by LY294002, suppression of mTOR signaling by rapamycin, and expression of AKT siRNA. Moreover, our study observed that radiation-induced VEGF-C expression was at the translational level but not the transcriptional level. The result is similar to the finding of Morfoisse et al. [31]*.* They demonstrated that transcription-independent but translation-dependent up-regulation of VEGF-C in hypoxia stimulates lymphangiogenesis in tumors.

## Conclusion

In conclusion, in NSCLC cells, radiation induced VEGF-C expression at least in part through activation of the PI3K/Akt/mTOR pathway. In addition to the tumor cell itself, the irradiated supernatant promoted HUVEC and LEC proliferation, which was inhibited by VEGF-C-siRNA expression in cancer cells. The clinical implication of this study is the critical need to suppress radiation-enhanced microenvironment changes possibly with VEGF-C inhibition during treatment.

## References

[1] Mountain CF (1997). Revisions in the international system for staging lung cancer. Chest.

[2] Auperin A, Le Pechoux C, Pignon JP (2006). Concomitant radio-chemotherapy based on platin compounds in patients with locally advanced non-small cell lung cancer (NSCLC): a meta-analysis of individual data from 1764 patients. Ann Oncol.

[3] Penland SK, Socinski MA (2004). Management of unresectable stage III non-small cell lung cancer: the role of combined chemoradiation. Semin Radiat Oncol.

[4] Perez CA, Bauer M, Edelstein S (1986). Impact of tumor control on survival in carcinoma of the lung treated with irradiation. Int J Radiat Oncol Biol Phys.

[5] Jeltsch M, Kaipainen A, Joukov V (1997). Hyperplasia of lymphatic vessels in VEGF-C transgenic mice. Science.

[6] Lymboussaki A, Achen MG, Stacker SA (2000). Growth factors regulating lymphatic vessels. Curr Top Microbiol Immunol.

[7] Saaristo A, Veikkola T, Enholm B (2002). Adenoviral VEGF-C overexpression induces blood vessel enlargement, tortuosity, and leakiness but no sprouting angiogenesis in the skin or mucous membranes. FASEB J.

[8] Arinaga M, Noguchi T, Takeno S (2003). Clinical significance of vascular endothelial growth factor C and vascular endothelial growth factor receptor 3 in patients with nonsmall cell lung carcinoma. Cancer.

[9] Kajita T, Ohta Y, Kimura K (2001). The expression of vascular endothelial growth factor C and its receptors in non-small cell lung cancer. Br J Cancer.

[10] Saintigny P, Kambouchner M, Ly M (2007). Vascular endothelial growth factor-C and its receptor VEGFR-3 in non-small-cell lung cancer: concurrent expression in cancer cells from primary tumour and metastatic lymph node. Lung Cancer.

[11] Jackowski S, Janusch M, Fiedler E (2007). Radiogenic lymphangiogenesis in the skin. Am J Pathol.

[12] Nathanson SD, Westrick P, Anaya P (1989). Relationship of spontaneous regional lymph node metastases to dose of local irradiation of primary B16 melanomas. Cancer Res.

[13] Talmadge JE, Wolman SR, Fidler IJ (1982). Evidence for the clonal origin of spontaneous metastases. Science.

[14] Heisel MA, Laug WE, Stowe SM (1984). Effects of X-irradiation on artificial blood vessel wall degradation by invasive tumor cells. Cancer Res.

[15] Cheng JC, Chou CH, Kuo ML (2006). Radiation-enhanced hepatocellular carcinoma cell invasion with MMP-9 expression through PI3K/Akt/NF-kappaB signal transduction pathway. Oncogene.

[16] Joukov V, Sorsa T, Kumar V (1997). Proteolytic processing regulates receptor specificity and activity of VEGF-C. EMBO J.

[17] Folkman J (1971). Tumor angiogenesis: therapeutic implications. N Engl J Med.

[18] Petrova TV, Makinen T, Makela TP (2002). Lymphatic endothelial reprogramming of vascular endothelial cells by the Prox-1 homeobox transcription factor. EMBO J.

[19] Shibuya K, Komaki R, Shintani T (2007). Targeted therapy against VEGFR and EGFR with ZD6474 enhances the therapeutic efficacy of irradiation in an orthotopic model of human non-small-cell lung cancer. Int J Radiat Oncol Biol Phys.

[20] Sonveaux P, Brouet A, Havaux X (2003). Irradiation-induced angiogenesis through the up-regulation of the nitric oxide pathway: implications for tumor radiotherapy. Cancer Res.

[21] Nozue M, Isaka N, Fukao K (2001). Over-expression of vascular endothelial growth factor after preoperative radiation therapy for rectal cancer. Oncol Rep.

[22] Dent P, Yacoub A, Contessa J (2003). Stress and radiation-induced activation of multiple intracellular signaling pathways. Radiat Res.

[23] Cordes N, Rödel F, Rodemann HP (2012). Molecular signaling pathways. mechanisms and clinical use. Strahlenther Onkol.

[24] Massion PP, Taflan PM, Shyr Y (2004). Early involvement of the phosphatidylinositol 3-kinase/Akt pathway in lung cancer progression. Am J Respir Crit Care Med.

[25] Kobayashi M, Nagata S, Iwasaki T (1999). Dedifferentiation of adenocarcinomas by activation of phosphatidylinositol 3-kinase. Proc Natl Acad Sci U S A.

[26] Sunavala-Dossabhoy G, Fowler M, De Benedetti A (2004). Translation of the radioresistance kinase TLK1B is induced by gamma-irradiation through activation of mTOR and phosphorylation of 4E-BP1. BMC Mol Biol.

[27] Albert JM, Kim KW, Cao C (2006). Targeting the Akt/mammalian target of rapamycin pathway for radiosensitization of breast cancer. Mol Cancer Ther.

[28] Dionysopoulos D, Pavlakis K, Kotoula V (2013). Cyclin D1, EGFR, and Akt/mTOR pathway. Potential prognostic markers in localized laryngeal squamous cell carcinoma. Strahlenther Onkol.

[29] Kobayashi S, Kishimoto T, Kamata S (2007). Rapamycin, a specific inhibitor of the mammalian target of rapamycin, suppresses lymphangiogenesis and lymphatic metastasis. Cancer Sci.

[30] Tang Y, Zhang D, Fallavollita L, Brodt P (2003). Vascular endothelial growth factor C expression and lymph node metastasis are regulated by the type I insulin like growth factor receptor. Cancer Res.

[31] Morfoisse F, Kuchnio A, Frainay CH (2014). Hypoxia induces VEGF-C expression in metastatic tumor cells via a HIF-1α-Independent translation-mediated mechanism. Cell Rep.

